# Perceived and Ideal Body Image in Young Women in South Western Saudi Arabia

**DOI:** 10.1155/2015/697163

**Published:** 2015-11-11

**Authors:** Atika Khalaf, Albert Westergren, Vanja Berggren, Örjan Ekblom, Hazzaa M. Al-Hazzaa

**Affiliations:** ^1^The PRO-CARE Group, School of Health and Society, Kristianstad University, 291 88 Kristianstad, Sweden; ^2^Department of Public Health Sciences, Karolinska Institute, 171 77 Stockholm, Sweden; ^3^Department of Health Sciences, Medical Faculty, Lund University, 221 00 Lund, Sweden; ^4^Åstrand Laboratory of Work Physiology, The Swedish School of Sport and Health Sciences, 114 86 Stockholm, Sweden; ^5^Pediatric Exercise Physiology Research Laboratory, College of Education and Obesity Research Chair, King Saud University, Riyadh 11451, Saudi Arabia

## Abstract

*Objectives*. The aim of this study was to investigate perceived and ideal body image (BI) and associated factors among female university students in Saudi Arabia. *Methods*. This cross-sectional study included 663 university female students. Anthropometric measurements including weight, height, BMI, and BI perception (the 9-figure silhouette) were obtained. Descriptive and logistic regression analysis were conducted. *Results*. An agreement between actual, perceived, and ideal BI was found in 23% of the participants. Behavioral (activity levels), social (presence of obese parents and fathers' level of education), and economic factors (households' monthly income, number of cars in the household, and kind of residence) were positively and significantly associated with the desire to be thinner. Similarly, socioeconomic associations (number of sisters and number of cars in the household) correlated positively and significantly with the desire to be heavier. *Conclusions*. The whole family should rather be considered in interventions related to appearance concerns and BI discrepancies. Furthermore, campaigns targeting improvement of adolescents' physical self-image should be a major priority of the public health sector.

## 1. Introduction

The prevalence of overweight and obesity in the Kingdom of Saudi Arabia (KSA) has increased in recent decades, with females (75–88%) generally having a higher prevalence rate than males (70–85%) [1]. While the health consequences of obesity in young adulthood are well recognized [2], body image (BI) problems are also much more common among overweight and obese people, sometimes leading to depressive symptoms and psychological distress [3]. Studies have shown that despite low rates of obesity among university students in general, many students, especially females, perceive themselves to be overweight [4]. This inappropriate weight perception is linked to unhealthy behaviors, including eating disorders [5].

Previous research has indicated that BI is a multidimensional concept that involves neurological, psychosocial, and cultural factors [6]. Intercultural differences clearly exist in BI norms. Data from the United States showed that African-American and Caucasian adults and adolescents appear to differ significantly in BI preference [7]. In addition, Fitzgibbon et al. [8] found that black and Hispanic women did not report BI discrepancy until they were overweight (BMIs of 29.2 and 28.5, resp.), while white women experienced BI discrepancy at a lower BMI level (BMI = 24.6). As to the BI preference among Arab women, research conducted on the Bahraini adult population focusing on lifestyle and social factors associated with obesity revealed that Arab women consider the midrange of fatness to be the most socially acceptable, while very thin or obese body sizes were least accepted [9]. The findings from one study on body weight perception, conducted in Dammam in the eastern province of KSA, have shown that many severely obese women considered themselves to be of normal weight [10]. In the same study it was also found that 30.6% of the participants (obese and nonobese women) chose a figure corresponding to “a little overweight” as the ideal female body size [10]. However, women with higher educational level were more likely to favor slimness as an ideal body shape [10]. A similar study investigated the social factors associated with body shape preferences for females and males as perceived by Arab women in Qatar and found a tendency for the participants to prefer an image of a midrange of body fatness [11]. The obesity rate among females in southern KSA is lower than in the rest of the country [12] but our knowledge about the perceived physical appearance in this population is scarce.

Furthermore, to the authors' knowledge no studies have attempted to link BI perceptions among healthy females in higher education with socioeconomic factors. Therefore, the targeted population in this study was university female students. One aim of this study was to investigate subjective BI, that is, perceived and ideal BI, among female university students in Saudi Arabia. A further aim was to examine the effect of possible socioeconomic and lifestyle factors on BI perceptions. Our hypothesis was that the most pronounced combination of discrepancies would be that of having a warped self-image (believing that one is either thinner or heavier than one actually is) while also having a discrepancy in subjective goal image (between perceived and ideal BI, wanting to be thinner or heavier than one perceives oneself to be). According to previous studies, the primary factor for the actual decision of attempting weight control in different age groups was not the actual body weight but the perception on the body weight of the self [13, 14]. Therefore, the subjective BI is in focus for this study.

## 2. Methods

This cross-sectional study involved college-aged females from a university in south western Saudi Arabia. The sample was selected using a multistage stratified random selection procedure. A total of 663 university students drawn equally from all four levels (freshman, sophomore, junior, and senior levels) were included in the sample. The study was approved by the Ethical Committee of King Khalid University, Abha, KSA, and informed consent was obtained from each participant prior to data collection.

The instrument used in this study for assessment of lifestyle habits and socioeconomic factors was a self-reported questionnaire including measures of socioeconomic factors, along with physical activities [15], building on the Arab Teens Life Style (ATLS) questionnaire [16]. The questionnaire included specific questions to determine the frequency, duration, and intensity of diverse forms of physical activities during a typical week. The different physical activities performed weekly were recorded. The energy expenditure in relation to household, leisure, transport, fitness, and sports activities was calculated and transformed into Metabolic Energy Turnover (MET) [17]. The total MET-min/week is achieved by multiplying the intensity of the different activities (in METs) by time spent on the activity (in minutes/week). For the activity levels, cut-off points that were based on tertiles of total activity energy expenditure, persons were considered as low active when they achieved ≤ 611 MET-min/week, moderate active 612 to 1390 MET-min/week, and high active ≥ 1391 MET-min/week [18].

Body weight (to the nearest 100 grams) and body height (to the nearest cm) were measured using a calibrated medical scale and a stadiometer, respectively. BMI was then categorized in accordance with WHO guidelines into Caucasian cutoffs: underweight (BMI < 18.49), normal weight (BMI 18.5–24.9), overweight (BMI 25–29.9), and obesity (BMI <30).

Information on the participants' perceived BI was obtained through nine illustrations of female body shapes ranging from very thin to morbidly obese, using the silhouettes scale developed by Stunkard et al. [19]. The 9-figure silhouette was validated in the study of Stunkard et al. [19] on a sample of 1000 adults. In another study the figure rating scale was also tested and showed good reliability for the ratings of “ideal figure,” “the figure subjects think they have,” and “the figure that reflects how they feel most of the time” (correlation coefficients for females = 0.71, 0.89, and 0.83, resp.) [20].

The participants were first asked to select the body shape they perceived their own bodies as being most like and then the ideal body shape they desired. The BMI was linked to the silhouetteses described by Stunkard et al. [19], here labelled as “actual BI.”

### 2.1. Statistical Analysis

In the analysis, we used categorization of discrepancies between the participants' perceived and ideal body shape. To test our hypothesis we cross-tabulated the warped self-image against discrepancy in subjective goal image.

From the variable “discrepancy between perceived and ideal BI,” two new variables were created. These two variables were “a desire to be thinner” (coded as 0 = satisfied and 1 = desires a thinner body than perceived) and “a desire to be heavier” (coded as 0 = satisfied and 1 = desires a heavier body than perceived). A logistic regression analysis (backward conditional) was then chosen to examine the association between each of the two subjective BI variables and behavioral and socioeconomic factors. The following variables were entered: age; marital status; number of sisters; number of brothers; fathers' level of education; mothers' level of education; number of cars in the household; kind of residence; households' monthly income; presence of obese parents; time spent in front of TV; time spent in front of computer; number of sleeping hours; activity levels based on METs tertiles; and occupational status of parents.

## 3. Results

The participants had a mean (SD) age of 20.4 (1.49) years and about 93% of them were unmarried. More than half (56.9%) of the participating students had a normal body weight, while the prevalence of underweight, overweight, and obesity was 19.3%, 17.8%, and 6.0%, respectively (Table 1).

In subjective goal image, discrepancies were found among the participants who wanted to be thinner (44.1%) or heavier (19.7%) than their perceived BI. Warped self-image existed in those who felt thinner (16.5%) or heavier (19.1%) than their actual BI and 5.9% of those who felt thinner than their actual BI desired a thinner body than their perceived one. Only 23.3% of the students had an agreement between their actual, perceived, and ideal BI. Of those with accurate perception of body shape, 25.5% wanted to be thinner than they perceived themselves and 15.6% wanted to be heavier than their perceived BI (Table 2).

The multiple logistic regression analysis (Table 3) revealed significant positive association between “a desire to be thinner” and activity levels (moderate compared to low activity, OR 1.74, *p* value 0.019), presence of obese parents (one obese parent compared to none, OR 2.70, *p* value 0.006), and fathers level of education (any higher education compared to primary or less, OR 1.69–2.16, *p* value 0.009–0.049). The economic factors associated positively with “a desire to be thinner” were monthly income (lowest and highest income compared to 5001–10000 SAR, OR 2.66, *p* value 0.035, and OR 1.93, *p* value 0.018, resp.), number of cars in the household (three cars or more compared to two cars, OR 1.57, *p* value 0.036), and kind of residence (traditional house compared to apartment, OR 3.58, *p* value 0.047).

Significant positive associations were identified between “a desire to be heavier” and number of sisters (four to five compared to none, OR 1.95, *p* value 0.026) and number of cars in the household (three cars or more compared to two cars, OR 1.86, *p* value 0.011) (Table 3).

## 4. Discussion

The main findings of the current study are (1) only 23.3% of the participants showed an agreement between their actual, perceived, and ideal body shape and (2) behavioral, social, and economic correlates significantly predicted the desire to be thinner and heavier. Our hypothesis was that there would not be a combination of discrepancies between having a warped self-image and a discrepancy in subjective goal image. The results of our study allow us to reject the null hypothesis since the results show a significant (Chi-square 41.8, *p* value < 0.0001) interaction between the two variables of warped self-image and discrepancy in subjective goal image.

In the present study, only 23.3% of the participants showed an agreement between their ideal, perceived, and actual BI, while 44.1% desired a thinner body shape than their perceived one. Our results are similar to other studies' findings, reporting a high proportion of normal weight women (61%) who desire a slimmer body weight in a Lebanese university population [21], (76%) in Korean students, and (73%) in Chinese students [22]. According to scientific studies covering this important area, the mass media are partly responsible for the high rates of BI disturbance and eating disorders among women [22–25]. Although many societies value a thin body for women [26], this ideal may be especially dangerous to women because some female images in the media may not be attainable without dieting and taking anorexic actions [27]. Furthermore, the female adolescent years are often associated with weight and shape concerns, as reported in western Europe and the USA [26], in which a thinner female body is considered desirable. Weight and shape concerns may result in skipping meals and eating disorders in this young population [26, 28]. Skipping meals and eating disorders in their turn are associated with negative health behaviors and unhealthy lifestyle such as tobacco, alcohol, and substance abuse [26, 28]. According to the objectification theory of Fredrickson and Roberts [29], self-objectification leads women to experience appearance anxiety and body shame. Women who place unnecessary stress on appearance are more likely to report negative psychological and health consequences [24] such as reduced body satisfaction and self-esteem [25, 30] as well as restrained or disordered eating [24]. Restraint and disordered eating are a concern in Western societies [24]. Studies are needed to explore the reasons for BI concerns in order to further understand the phenomenon. With such knowledge a critical discussion about BI ideals in relation to media might possibly change the prevailing discourse about the “slim ideal.”

One of the most important findings of the current study is the positive association between the behavioural correlate (activity levels) with the desire to be thinner. Our findings can be confirmed by previous studies' where significant associations between girls and young women's lifestyle and behavioural factors and BI concerns were reported [31–33]. As an example, the study of Kirkcaldy et al. [32] reported that adolescents who engaged in regular physical activities were characterised by lower BI concerns. On the other hand, Slater and Tiggemann [31] conclude that the BI concerns may contribute to adolescent girls' reduced rates of participation in sports and other physical activities. Our results highlight young women's and adolescents' efforts to become thinner through exercise. However, we cannot state that this is a healthy behaviour since the findings are related to subjective BI, not actual BI. This should be the focus for future studies.

This study's findings showed that a high percentage (19.7%) of the participants wanted to be heavier than their perceived BI. This may be due to the desire to meet the local social requirements for the acceptable body size for women. In Arab societies, curvaceousness has traditionally been considered a characteristic of feminine beauty [21]. According to Musaiger et al. [11] being a little overweight is the most socially accepted body size in Saudi society. A more recent study conducted in Riyadh [34] among 799 university female students showed that 17.4% of the obese participants and 54.2% of the overweight participants perceived themselves as of normal weight. Another current study focusing on attractive female body weight and female body dissatisfaction in 26 countries across 10 world regions [35] showed that, in places with low socioeconomic status heavier bodies were still preferred, which is not the case in KSA, which is ranked among the high socioeconomic places. If it is true that the participants in the present study wanted to gain weight to meet social requirements, this leads to the next question about the objectification theory [29]: do university aged female students try to obtain social acceptance through objectification of their bodies? Further, probing research on the subject of social requirements and the BI perception is needed, with special focus on overweight and obesity.

Although this study offers insight into the current perception of weight status in a healthy sample in higher education, it has a number of limitations. Generalizability is limited because of the selectivity of the study setting and the restriction to one university. On the other hand, the setting was a governmental university with selection of students from different socioeconomical and ethical groups. Another potential shortcoming of this study might be the rigor with which its results can be applied to perception of BI by the entire Saudi population because the study participation was limited to female university students. A relevant query related to the study's finding of BI, namely, if BI discrepancy was due to response bias or if it was indicative of less certainty in self-perception, could have been tested in a test-retest consistency of responding. This has, however, not been done. On the other hand, the strengths of the study could be that the number of participants was considered representative of the studied female university students, the used protocol is a reproducible and validated questionnaire [13], and the study's procedures were highly standardized. Furthermore, the questionnaire is comparable to other self-reporting instruments on the whole. Therefore, the results of this study could be generalizable to other female university students, not only in KSA but also in other Arab countries.

## 5. Conclusions

Discrepancies between perceived and ideal BI are associated with social and economic factors. However, it cannot be ascertained whether these discrepancies are due to psychological or physical factors. It depends on whether perceived BI in these women is relevant to actual BI. This should be the focus for future studies. Interventions related to appearance anxiety and BI discrepancies considering the whole family should, therefore, be applied. The discrepancies among KSA female students could possibly lead to eating disorders and thereby increased prevalence of malnutrition, that is, underweight or overweight and obesity. Studies on BI perception could be sought as essential tools for guiding the implementation of strategies for the promotion of healthy lifestyle and for providing the impetus for subsequent interventional studies to target BI discrepancies in all age groups of the female population in KSA. Furthermore, campaigns targeting improvement of adolescent self-image could be recommended to the public health sector.

## Figures and Tables

**Table 1:** Characteristics of the participants.

	*n* = 660
Age, years, mean (SD)	20.4 (1.49)
Minimum–maximum	18–25
Marital status, *n* (%)	
Not married	614 (92.6)
Married, without children	30 (4.5)
Married, with children	19 (2.9)
Weight status, *n* (%)^*∗*^	
Underweight 18,49 and under	127 (19.3)
Normal weight 18.5–24.99	376 (56.9)
Overweight 25–29.99	117 (17.8)
Obesity 30 and over	40 (6.0)

^*∗*^Weight status categorized according to WHO Caucasian BMI specifications.

**Table 2:** A cross-tabulation of the warped self-image (discrepancy between actual and perceived body image) and discrepancy in subjective goal image (between perceived and ideal body image), presented in frequencies and percentages. The categories of the discrepancies show that an agreement between the actual, perceived, and ideal body image is met in the middle of the table, that is, the value 147 (23.3%) of all cases. All other values report a kind of discrepancy in body image.

		Discrepancy between actual and perceived body image (warped self-image)			Total
		Feels thinner than one actually is	Accurate body image	Feels heavier than one actually is	
Discrepancy in subjective goal image, that is, between perceived and ideal body image	Wants to be thinner than perceived body image	37 (5.9)	161 (25.5)	80 (12.7)	278 (44.1)
		“Inaccurately feels thin and wants to be thinner”	“Accurate body image and wants to be thinner”	“Inaccurately feels heavy and wants to be thinner”	
	Current body image is ok	44 (7.0)	147 (23.3)	37 (5.9)	228 (36.2)
		“Feels thin and satisfied”	“Accurate body image and satisfied”	“Feels heavy and satisfied”	
	Wants to be heavier than perceived body image	23 (3.6)	98 (15.6)	3 (0.5)	124 (19.7)
		“Inaccurately feels thin and wants to be heavier”	“Accurate body image and wants to be heavier”	“Inaccurately feels heavy and wants to be heavier”	

Total		104 (16.5)	406 (64.4)	120 (19.1)	630 (100)

Pearson Chi-square 41.8 (df = 4), *p* value < 0.0001.

**Table 3:** Results from the multiple logistic regression analysis (backward conditional) for “a desire to be thinner” (*n* = 525) and “a desire to be heavier” (*n* = 370). Only variables with statistical significance (*p* value < 0.05) are presented here.

	OR	95% CI		*p* value
A desire to be thinner (0 = satisfied and 1 = desires a thinner body than perceived)				
Activity levels				
Low active				0.037
Moderately active	1.74	1.096	2.757	0.019
High active	1.07	0.682	1.682	0.766
Presence of obese parents				
None of the parents is obese^*∗*^				0.001
Mother or father is obese	2.70	1.333	5.459	0.006
Both parents are obese	1.48	0.718	3.060	0.287
Fathers' level of education				
Primary or less^*∗*^				0.037
Primary higher	1.89	1.001	3.555	0.049
Secondary	2.16	1.216	3.826	0.009
Bachelor or higher	1.69	1.019	2.789	0.042
Households' monthly income				
5001–10 000 SAR^*∗*^				0.004
3000 SAR or less	2.66	1.073	6.573	0.035
3001–5000 SAR	0.96	0.521	1.778	0.902
10 001–15 000 SAR	0.82	0.469	1.446	0.499
More than 15 000 SAR	1.93	1.120	3.308	0.018
Cars in the household				
Two cars^*∗*^				0.067
One car or none	0.91	0.410	2.012	0.813
Three cars or more	1.57	1.029	2.390	0.036
Kind of residence				
Apartment^*∗*^				0.108
Villa	1.87	0.512	6.852	0.343
One floor in villa	3.10	0.904	10.642	0.072
Traditional house	3.58	1.020	12.554	0.047

A desire to be heavier (0 = satisfied and 1 = desires a heavier body than perceived)				
Number of sisters				
None^*∗*^				0.144
Only one	2.05	0.577	7.256	0.267
Two-three	0.91	0.389	2.123	0.825
Four-five	1.95	1.082	3.524	0.026
Six or more	1.52	0.867	2.670	0.143
Cars in the household				
Two cars^*∗*^				0.037
One car or less	1.36	0.533	3.444	0.524
Three cars or more	1.86	1.156	3.001	0.011

^*∗*^Reference category for further statistical analysis.

OR: odds ration and CI: confidence interval.

Entered variables: age; marital status; number of sisters; number of brothers; fathers' level of education; mothers' level of education; number of cars in the household; kind of residence; households' monthly income; presence of obese parents; time spent in front of TV; time spent in front of computer; number of sleeping hours; activity levels based on METs tertiles; and occupational status of parents.
